# Spatiotemporal clustering analysis of Expanded Program on Immunization (EPI) vaccination coverage in Pakistan

**DOI:** 10.1038/s41598-020-67839-0

**Published:** 2020-07-03

**Authors:** Muhammad Farooq Umer, Shumaila Zofeen, Wenbiao Hu, Xin Qi, Guihua Zhuang

**Affiliations:** 1 School of Public Health, Xi’an Jiaotong University Health Science Center, Xi’an, 710061 China; 2 School of Public Health and Social Work, Queensland University of Technology, Kelvin Grove, QLD 4059 Australia; 3 Global Health Institute, Xi’an Jiaotong University Health Science Center, Xi’an, 710061 China

**Keywords:** Health care, Medical research

## Abstract

Universal vaccination coverage is still far from desired targets in many global regions including Pakistan, despite the success stories and its scientifically proven benefits. EPI Pakistan vaccination coverage data 2012–2016, at district level was collected from Federal EPI Pakistan. District-wise population data were collected from Pakistan Bureau of Statistics. Descriptive statistics and sequence plots were performed in SPSS 13.0. Purely spatial scanning analysis was done in SaTScan 9.4.4 using discrete Poisson model for detection of low vaccination coverage clusters. Geographical information system (GIS) was used to display spatial patterns and clusters of low vaccination coverage districts in Pakistan. Average annual EPI vaccination coverage in each study year were; 70.98 in 2012, 69.39% in 2013, 66.74% in 2014, 61.47% in 2015, and 67.01% in 2016, respectively. Cumulative average national vaccination rate (2012–2016) for all types of EPI vaccines was 60.60%. Average national vaccination rate for BCG, OPV3, pentavalent3 and measles1 was 67.12%, 58.53%, 58.47%, and 58.29%, respectively. Spatial cluster analysis demonstrated that most of low coverage districts for BCG, OPV3 and pentavalent3 were from FATA and KPK; while measles1 low coverage districts belonged to Balochistan. Future research should probe factors involved in low vaccination coverage in high risk districts.

## Introduction

The invention of and advancements in the childhood vaccination has revolutionized human healthcare and facilitated to averting morbidity and mortality from many vaccine-preventable infectious diseases^1^. Immunization not only has helped preventing life-threatening diseases but also benefitted mankind by increasing their life expectancy (life span has increased by 15 to 25 years since the commencement of vaccines) and in improving quality of life^2,3^. However, these successes cannot mask the failures in shape of millions of deaths of the unvaccinated children annually, across the globe^4^. Estimated 1.5 million children died of vaccine-preventable diseases in each of 2013 and 2017 respectively which construes that the world could have avoided an additional huge number of deaths a year had the universal immunization coverage achieved^5,6^.

Despite the fact that overall vaccination coverage in 2018 remained around 86%^6^, there remained a wide disparity between different World Health Organization (WHO) regions, e.g., the Americas, Europe and Western Pacific maintained over 90% immunization coverage of DTP3 containing vaccine whilst these rates were low for most of the countries in Africa and some in Asia^7^. Low levels of immunization coverage in the low-and middle-income countries pose a serious challenge to achieving universal vaccination coverage goals^8,9^. For any child to develop adequate immunity against the particular disease, adherence to complete vaccination schedule is of critical importance, as, incomplete vaccination leads to partial immunity and the disease risk persists^10,11^. There have been fatal outbreaks of vaccine-preventable infectious diseases in the developing countries which reflect the presence in large numbers of non-and under-vaccinated children^12,13^. As a matter of fact, the bottommost ten countries with lowest vaccination coverage belonged to either low or lower-middle income group^7^. Approximately 19.4 million eligible children did not receive DTP3 dosage worldwide—around 40% (8 million) of these children lived in war-afflicted areas^6^.

In Pakistan, government patronage for vaccination coverage through the Expanded Programme on Immunization (EPI) has been there for decades yet the low coverage areas have been stubbornly in existence across the country^14^. This has increased vulnerability to vaccine preventable diseases and not surprisingly Pakistan has been amongst countries with highest mortality and morbidity rates in children under 5 years of age^15,16^. Under-5 deaths make nearly half of all the deaths in Pakistan, which is around 8 to 10% of all deaths in the developed countries^17^. Pakistan has the lowest vaccination coverage statistics amongst the sub-continent countries (Bangladesh, Sri Lanka and India)^18^. These neighbouring countries had also overcome their respective indigenous polio transmission since 2011 through their massive scale high quality vaccination campaigns^18–20^, unlike Pakistan which is still struggling with Polio endemicity (along with Afghanistan and Nigeria)^21^.

Statistics from different sources in Pakistan have reported varying percentages of vaccination coverage, ranging from 40 to 80% with wide variations between provinces as well as between male and female children^22^. The higher percentages for vaccination coverage are generally reported by the government while some researchers and non-governmental agencies have reported low vaccination coverages across Pakistan over the time^23–25^. This variation may be attributed to the methodology adopted in collection of data (recall/record base data, estimation of the numerators/denominators), over/miss-reporting of the collected data, training/skill of the field teams and some other socio-geographical factors (parents’ knowledge, religious beliefs and accessibility to health facility).

EPI is virtually the exclusive provider of immunization services in Pakistan, conducting around 97% of the total immunization activities in the country. EPI in Pakistan vaccinates more than 5 million children under 1 year of age annually, in order to provide them protection against eight vaccine preventable infectious diseases through its routine immunization services^26^. Vaccination coverage with third dose of diphtheria, pertussis and tetanus (DTP3) is a commonly used indicator for the routine immunization services’ performance of various countries; whereas, Bacillus Calmette Guerin (BCG), third dose of oral polio vaccine (OPV3), pentavalent3 (containing DTP3) and Measles1 are considered to be yardstick of completed vaccination schedule^27^. Therefore, this study did not discuss rest of the vaccines which are included in EPI Pakistan routine immunization schedule but are further than the criteria of labelling a child as fully immunized (e.g. for *Haemophilus Influenzae*, Meningitis, Rota virus, Pneumonia and 2nd dose of Measles).

Epidemiologists have been interested in the geographical clustering of diseases^28^ and other public health problems such as communities with non/low vaccination^29^. Low vaccination coverage areas tend to exist in clusters and spatial scan statistics are an important tool to detect them for their subsequent targeting by the concerned authorities^29^. There are only few studies available on vaccination coverage discussing country-wide data and even scarce studies using modern statistical methods (e.g., spatial analysis) in Pakistan. The available data on vaccination coverage in Pakistan is limited and on many occasions faulty and over-estimated^30^. The available studies on vaccination coverage either discuss secondary data from surveys across Pakistan or are about one or few districts and predominantly about single antigen^25,31–33^. Our study has tried to contribute in these lacking areas of spatial analysis on vaccination coverage across Pakistan.

## Results

Total number of children vaccinated in each year (2012 to 2016) and for each vaccination type are shown in Table 1.

**Table 1:** Number of vaccinated children for each EPI vaccination type in Pakistan, 2012–2016.

Vaccination type	2012	2013	2014	2015	2016
BCG	6,002,983	6,093,216	6,087,530	5,825,405	6,217,308
OPV3	5,307,855	5,365,150	5,340,132	5,243,034	5,370,649
Pentavalent3	5,255,236	5,378,442	5,338,499	5,213,164	5,363,685
Measles1	5,428,966	5,602,642	5,354,079	5,185,488	5,444,831

### Descriptive analysis

The descriptive statistics were indicated in Table 2. In this table, high standard deviation and significant difference between minimum and maximum values indicate widely spread out vaccination coverage status among Pakistan districts. The coverage rates with highest mean values for all the vaccine types were found in 2012, while the lowest mean vaccination coverage rates were seen in 2015, e.g., mean BCG vaccination coverage rate was 70.89 in 2012, which was highest in all the study years, whereas, in 2015 it was 61.79 and lowest of all the study years.

**Table 2:** Summary of descriptive statistics of EPI vaccination coverage rates (per 100) by type at the district level in Pakistan, 2012–2016.

Year	Vaccine type	Mean	Std. deviation	Minimum	Quantiles			Maximum
					25	50	75	
2012	BCG	70.89	15.94	24.53	62.52	73.77	80.64	98.02
	OPV 3	62.38	14.73	17.56	56.61	67.11	71.21	93.62
	Pentavalent 3	62.09	14.58	17.56	56.58	65.81	70.51	93.62
	Measles 1	62.17	16.16	15.80	54.16	66.55	74.75	87.93
2013	BCG	69.34	15.18	8.02	63.35	73.07	78.94	97.69
	OPV 3	60.29	15.70	3.98	55.31	65.53	70.52	96.23
	Pentavalent 3	60.55	15.29	5.34	55.31	65.56	70.63	96.23
	Measles 1	61.46	17.29	6.10	52.40	67.16	72.94	95.34
2014	BCG	66.83	15.81	10.98	59.93	71.46	76.70	97.87
	OPV 3	57.46	16.25	1.27	51.31	62.78	70.08	92.70
	Pentavalent 3	57.47	16.18	3.82	51.34	62.78	70.10	92.70
	Measles 1	57.18	16.82	5.68	47.43	61.76	70.46	80.51
2015	BCG	61.79	16.10	3.55	54.47	66.12	73.23	95.14
	OPV 3	55.83	16.11	1.77	48.47	60.97	67.48	85.79
	Pentavalent 3	55.63	15.94	1.77	48.47	60.97	66.99	85.79
	Measles 1	54.48	17.57	2.51	42.52	59.78	68.33	84.48
2016	BCG	67.14	14.37	18.19	57.48	71.02	76.70	97.47
	OPV 3	57.61	14.56	5.83	49.32	61.42	68.11	87.60
	Pentavalent 3	57.57	14.54	4.82	49.32	61.42	67.77	87.60
	Measles 1	57.55	16.33	7.50	47.28	61.72	69.92	92.39

### Spatiotemporal and spatial clustering analysis

**Figure 1:**
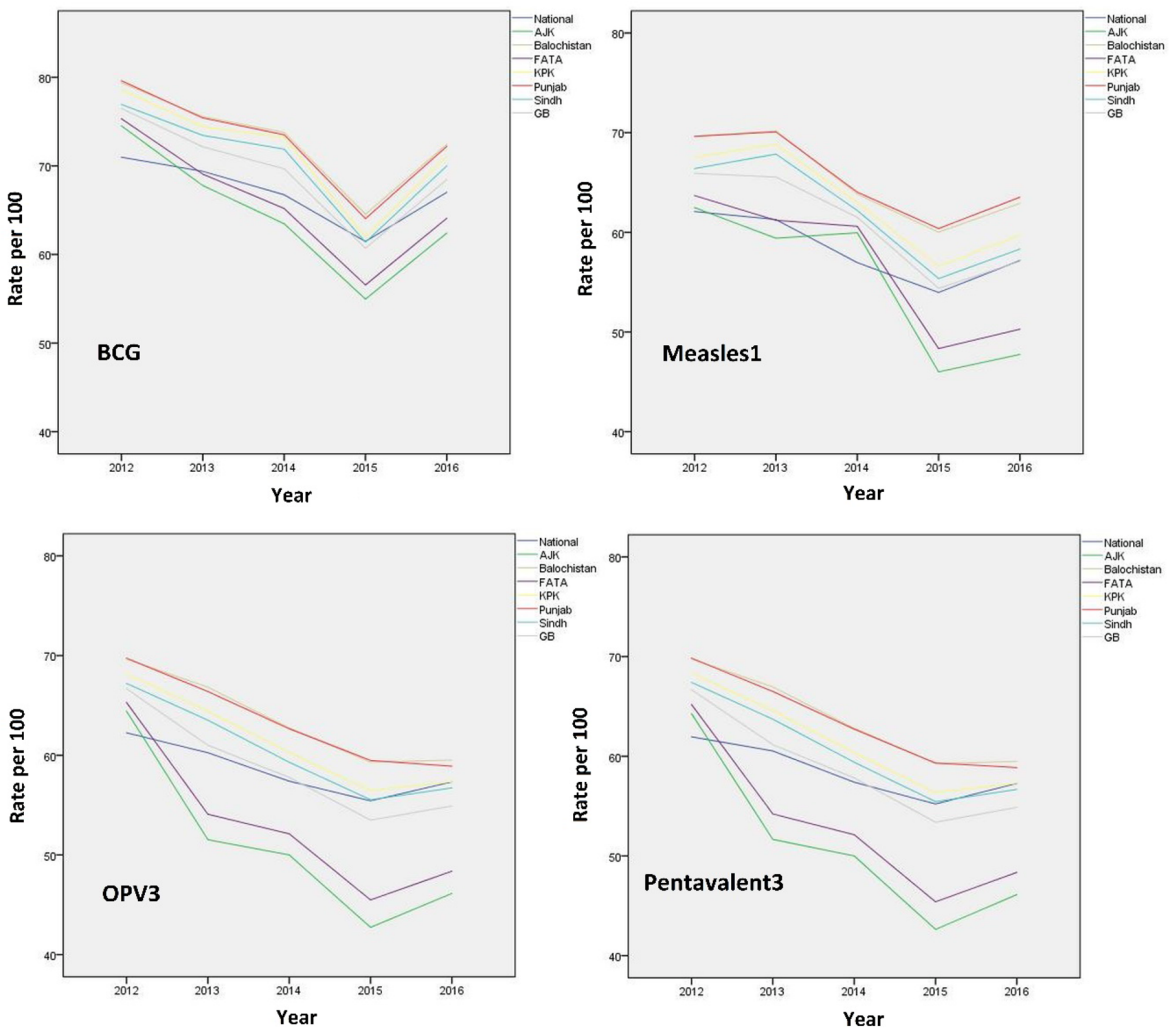
Sequence plot for EPI vaccination rates by type at national and provincial level in Pakistan, 2012–2016.

The sequence plot for vaccination rates per 100 children for each type of vaccination were shown in the Fig. 1 for national level as well as for all the provinces in Pakistan. The figure indicated that the rates (percentage) for BCG vaccination coverage were highest amongst all types of vaccination and there was a decline in 2015 in the rates of all types of vaccination coverage at the district level in Pakistan.

**Figure 2:**
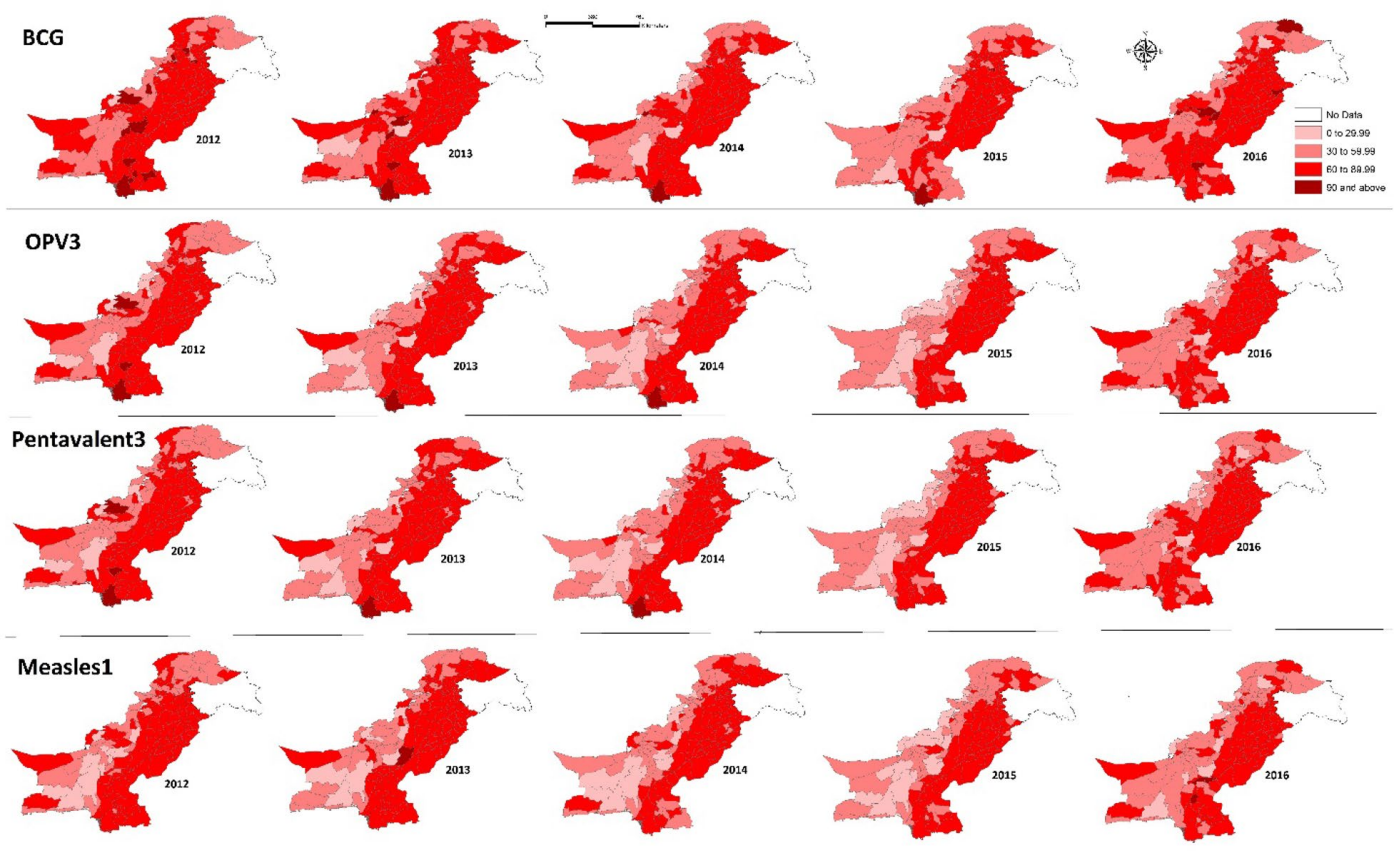
EPI vaccination coverage rates by type per 100 children at the district level in Pakistan, 2012–2016.

Figures 2 exhibited vaccination coverage rates per 100 children for all EPI vaccination types in Pakistan at the district level. Vaccination coverage rates for BCG were higher than the rest of the vaccination types and mostly lied in high to very high categories. The rates for OPV3 and pentavalent3 were similar to one another, as, according to the EPI vaccination schedule, both these types are administered at the same time. The vaccination coverage rates for BCG, OPV3 and pentavalent3 were found to be relatively higher in Azad Kashmir (north), Punjab (east) and most districts of Sindh (southeast), as compared to the rest of the country. The coverage rates for measles1 were found low to very low in most districts of Balochistan, Khyber Pakhtunkhwa (KPK) and Federally Administered Tribal Areas (FATA), especially in 2015.

Table 3 described the primary clusters for EPI low vaccination coverage by type in each of the study year at the district level in Pakistan. The yearly information on primary clusters including; radii of clusters, target population, vaccination rates per 100,000 children and the *P* values showing statistical significance were highlights of this table.

**Table 3:** Summary of primary high-risk clusters’ information of Expanded Program of Immunization (EPI) annual vaccination coverage by type at the district level in Pakistan, 2012–2016.

Type of vaccine	Year	Primary cluster radius (km)	Primary cluster target population	Number of vaccinated children in primary cluster	Vaccination rate/100,000	RR	*P* value
BCG	2012	123.33	229,703	126,337	54,886.3	0.72	< 0.0001
	2013	0	23,242	1,865	8,029.6	0.11	< 0.0001
	2014	123.33	241,469	115,953	48,051.7	0.65	< 0.0001
	2015	76.77	70,464	13,300	18,887.4	0.27	< 0.0001
	2016	99.71	112,512	43,863	38,904.4	0.54	< 0.0001
OPV3	2012	87.22	165,170	71,099	42,956.9	0.64	< 0.0001
	2013	0	23,242	925	3,982.5	0.6	< 0.0001
	2014	87.22	173,977	55,602	31,980.6	0.49	< 0.0001
	2015	91.38	73,710	10,724	14,558.6	0.23	< 0.0001
	2016	99.71	112,512	24,491	21,722.4	0.35	< 0.0001
Pentavalent 3	2012	87.22	165,170	71,577	43,245.7	0.65	< 0.0001
	2013	0	23,242	1,240	5,338.7	0.8	< 0.0001
	2014	87.22	173,977	56,208	32,329.2	0.5	< 0.0001
	2015	91.38	73,710	10,726	14,561.3	0.24	< 0.0001
	2016	99.71	112,512	24,011	21,296.6	0.34	< 0.0001
Measles 1	2012	199.41	179,754	82,847	45,993.7	0.67	< 0.0001
	2013	153.65	96,704	28,279	29,262.3	0.42	< 0.0001
	2014	180.86	865,089	420,780	48,672.4	0.75	< 0.0001
	2015	195.57	261,951	94,192	35,981.8	0.58	< 0.0001
	2016	191.94	1,454,135	731,131	50,175.4	0.77	< 0.0001

The size of primary clusters for measles1 low coverage were much larger than those for the other vaccination types included in the study, e.g., in 2014 and 2016 measles 1 clusters comprised of 22 and 29 districts, respectively; while for OPV3 and pentavalent3 vaccination types, there were seven districts per cluster in each of the corresponding study year. The measles1 clusters were also inconsistent and comprised of districts from different provinces for different years in the study.

**Figure 3:**
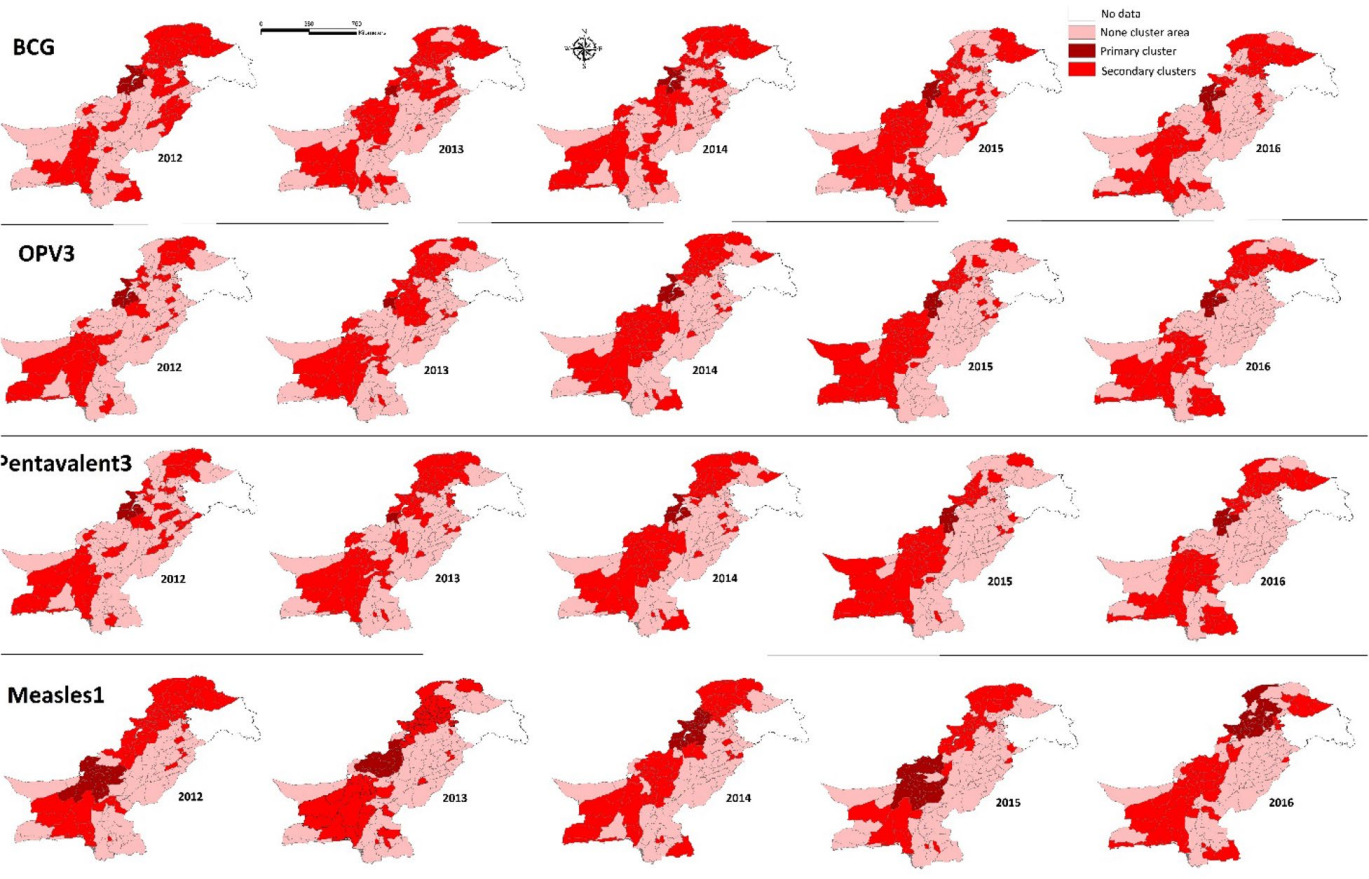
EPI low vaccination coverage by type clusters at district level in Pakistan, 2012–2016.

Figure 3 represented the primary, secondary and no cluster areas of low vaccination coverage clusters for all vaccination types in each year of study at the district level.

The districts detected in the primary clusters in each year of the study and for each type of vaccinations were as follows.

Table 4 depicts province wise details of the most significant clusters at the district level in Pakistan during the study period. Districts in low vaccination coverage primary clusters for BCG, in 2012, (total 10 districts, 5 each in FATA and KPK); in 2013, (1 district in FATA); in 2014, (total 10 districts, 5 each in FATA and KPK); in 2015, (total 5 districts, 3 in FATA, 1 each in KPK and Balochistan); in 2016, (total 7 districts, 4 in FATA, 2 in KPK and 1 in Balochistan).

**Table 4:** Province-wise distribution of primary high-risk clusters of Expanded Program of Immunization (EPI) annual vaccination coverage by type at the district level in Pakistan, 2012–2016.

Type of vaccine	Year	Total number of districts in primary clusters	Number of districts in FATA	Number of districts in KPK	Number of districts in Balochistan	Number of districts in Sindh	Number of districts in Punjab	Number of districts in GB/federal capital	Number of districts in AJK
BCG	2012	10	5	5	–	–	–	–	–
	2013	1	1	–	–	–	–	–	–
	2014	10	5	5	–	–	–	–	–
	2015	5	3	1	1	–	–	–	–
	2016	7	4	2	1	–	–	–	–
OPV3	2012	7	4	3	–	–	–	–	–
	2013	1	1	–	–	–	–	–	–
	2014	7	4	3	–	–	–	–	–
	2015	6	4	1	1	–	–	–	–
	2016	7	4	2	1	–	–	–	–
Pentavalent 3	2012	7	4	3	–	–	–	–	–
	2013	1	1	–	–	–	–	–	–
	2014	7	4	3	–	–	–	–	–
	2015	6	4	1	1	–	–	–	–
	2016	7	4	2	1	–	–	–	–
Measles 1	2012	13	–		13	–	–	–	–
	2013	9	2	–	7	–	–	–	–
	2014	22	11	10	–	–	1	–	–
	2015	18	–	–	18	–	–	–	–
	2016	29	7	20	–	–	1	1	–

Districts in low vaccination coverage primary clusters for OPV3 and pentavalent3 were alike for each of the corresponding years, i.e., in 2012, (total 7 districts, 4 in FATA and 3 in KPK); in 2013, (1 in FATA); in 2014, (total 7 districts, 4 in FATA and 3 in KPK); in 2015, (total 6 districts, 4 in FATA and 1 each in KPK and Balochistan); in 2016, (total 7 districts, 4 in FATA, 2 in KPK and 1 in Balochistan).

Districts in low vaccination coverage primary clusters for Measles 1: in 2012; (all 13 districts in Balochistan); in 2013 (total 9 districts, 7 in Balochistan and 2 in FATA) in 2014; (total 22 districts, 11 in FATA, 10 in KPK and 1 in Punjab); in 2015; (all 18 districts in Balochistan); in 2016; (total 29 districts, 20 in KPK, 7 in FATA and 1 each in Punjab and Federal capital).

## Discussion

This study explored EPI immunization coverage of the children vaccinated against vaccine preventable diseases, including Childhood Tuberculosis (BCG), Poliomyelitis (OPV3), Diphtheria, Pertussis, Neonatal Tetanus, *Haemophilus Influenzae* type B, Hepatitis B (pentavalent3 which contains DTP3) and Measles (Measles 1) over a period of 5 years (2012 to 2016) at the district level in Pakistan. During the study period, 30,226,442 children were vaccinated with BCG, 26,626,820 children were vaccinated with OPV3, 26,548,979 were vaccinated with pentavalent3 and 27,016,006 were vaccinated with measles1 vaccine throughout the country, respectively.

The spatial cluster analysis showed that most of the low vaccination districts for BCG, OPV3 and pentavalent3 were from FATA and KPK; while for measles1 vaccination, most of the detected districts belonged to Balochistan Province. There was a consistent pattern of distribution of these clusters, especially for OPV3 and pentavalent3 and in some years of study for BCG as well. However, measles1 clusters were inconsistently distributed in different years of study and over larger areas, especially those found in Balochistan. Only two districts from Punjab were detected for measles1 primary clusters one each in 2014 and 2016 respectively. However, none of the districts from Punjab, Sindh, Gilgit Baltistan and Azad Kashmir were seen in any of the primary spatial clusters of low vaccination coverage during the study period.

These findings were consistent with country wide surveys in Pakistan. A policy brief by “Research and Development Solutions” in 2012 stated that routine coverage rates for fully immunized children were significantly low in Pakistan, especially in FATA, KPK, Balochistan and Sindh, moreover the study also agreed that one in every five children remains unimmunized at the national level while in the rural population this rate is even higher with two out of every three children being non-immunized^17^.

Herd immunity is defined as the resistance a population develops against the spread of an infectious disease resulted from adequately high proportion of individuals being immune to that disease—especially through vaccination. There are different vaccination levels required to achieve herd immunity for various diseases, e.g. the herd immunity thresholds for Vaccine-Preventable Diseases are; Tuberculosis (BCG) not known, Polio (OPV) 50–95%, Diphtheria 83–85%, Tetanus N/A, Pertussis 80–94% (DTP) and Measles 92–94%^34^. Our study found that, at the national level, average vaccination rates (all types of vaccination, BCG, OPV3, pentavalent3, measles1) per 100 children were 60.60, 67.12, 58.53, 58.47 and 58.29 respectively. Whereas, according to previous surveys, average vaccination rate for each type of vaccination varied significantly in assessments done by different bodies. For example, the latest Multiple Indicator Cluster Surveys (MICS) of Punjab (63.8% male, 64.4% female)^35^, Sindh (43.1% male, 43.4% female)^36^, KPK (55.5% male, 55.5% female)^37^ and Balochistan (3.5% male, 4.8% female)^38^, and Pakistan Social and Living Standards Measurement Survey (PSLM 2014–2015) Punjab (70% male, 70% female), Sindh (45% male, 45% female), KPK (60% male, 56% female) and Balochistan (30% male, 25% female) clearly depict marked variation in the vaccination coverage statistics across different provinces in Pakistan. Punjab is the most populous province harbouring more than 50% of total population in Pakistan, followed by Sindh which is the second largest. Despite that our study showed better vaccination coverage statistics for both these provinces as compared to FATA, Balochistan and KPK, yet the number of non/under-vaccinated children are still very high owing to the size and density of their population; suggesting that the decision makers should not lose focus on them. Also some antigens showed similar statistics as in our study, e.g. average vaccination rate per 100 children for BCG was 88, 85 and 89, according to PSLM 10–11, 12–13 and 14–15 respectively^39–41^; and 85 and 88 according to PDHS 12–13 and 17–18 respectively^42,43^. BCG coverage was the highest among all the vaccination types and measles1 coverage was the lowest; similar trends were observed in some of the preceding assessments. However, there were varying trends in some of the districts and provinces in different years of this study.

Another study showed marked variation from our results with only 31% fully vaccinated children in Pakistan which is alarming and at the same time necessitate ponder over^24^. This huge discrepancy in the reported vaccination coverage status through different sources creates doubt on the health of data. Reporting bias in administrative data can be one of these factors as WHO and United Nations Children Fund (UNICEF) have already raised questions on the quality of administrative data reported by various member countries^44^. The numerator errors/bias (errors/bias in reporting number of doses administered by health staff either inadvertently or purposely to magnify their performance) has been accounted for in this study to a fair extent but there are other issues like wrong denominator estimations due to varying population growth rates in the districts and numerator/denominator mismatches due to migrations in and out of the districts and provinces which might have affected the results to some extent. Majority of the population in Pakistan live in the rural areas with urbanization trends increasing with the passage of time. Migration within the country is common, e.g. one third of these migrants moved from rural to the urban settings^45^. According to Pakistan Labour Force Survey 2014–2015, rural to urban migration accounted for 22.5% of the total flow of migrants within and between districts and out of these migrations, 43.8% in Balochistan, 25.1% in Punjab, 21.1% in KPK and 13.9% in Sindh^46^. These migration trends may explain the low vaccination coverage due to more missed children in Balochistan and higher vaccination trends in Sindh^23,47^. Also, the heterogeneities in coverage of different districts and provinces is a key factor in non-achievement of desired periodical targets and eventually of herd immunity at the subnational and national levels. Spatial heterogeneity in vaccination coverage impacts the health status of the residents as it can delay disease elimination, not only in the developing but also in the developed countries where nation-wide vaccination coverage rates are usually higher^48,49^.

All the national surveys (e.g., PSLM, Pakistan Demographic and Health Survey (PDHS) conducted in the past used similar measures and methods to estimate immunization coverage rates and yet produced varying results^40–43^. This was because of relying on recall of mother’s memory (as done in PDHS) or any one of the household member’s memory (as done in PSLM) for the estimation. On the other hand, assessment on the basis of child’s vaccination card record has resulted in underestimation because of poor record keeping practices by the household members^50^. In fact, both these methods (record based and recall based) cannot be relied upon independently, as they provided inaccurate estimation of the immunization rates^7^. This was confirmed by a study done in one district of Sindh where the accuracy of BCG vaccination coverage was assessed using mothers’ recall, vaccination card record and the record maintained by health staff (Vaccinator) at the healthcare facility. Mothers’ recall was moderately accurate, while vaccination card record and health staff record were very less accurate^51^. The vaccination coverage record maintained at respective healthcare facilities in Pakistan is often deficient as it usually reflects aggregated number of the vaccinated children only and lacks demographic information (e.g. name, age, sex and home address) of individual children. The vaccination process, however is carried out through a well versed healthcare system administered by trained staff with monitoring and evaluation being a part and parcel of it having varied efficiencies and capacities across the districts in Pakistan^52^. Hence in the current scenario it is the best available data to perform country wide analysis and do the forecasting for future vaccination requirements.

Besides Balochistan, very low immunization coverage statistics were also observed in FATA and KPK in our study, which is not a surprise. A few districts from Balochistan and KPK while most districts from FATA have been conflict struck with compromised law and order situations which resulted in humanitarian crises; millions of temporarily displaced persons, lack of basic equipment and professional staff in healthcare facilities, and poor access to health services. And this all added up to already lower immunization coverage status in these provinces^21,53,54^. In 2016 only, an estimated 20% of households in FATA did not vaccinate their children for any routine EPI vaccine^21^.

Formal registrations of the new-borns in Pakistan has not been widely practiced, but the trends have changed over the years, especially after the establishment of National Database Authority Pakistan (NADRA) in 2000. The registrations of the children increased between the ages of 2 to 4 as the birth certificates during the school admissions are mandatory, but there is much room of improvement in the current practices of child registration at birth^42^, which can contribute in bettering the vaccination coverage in the country.

The performance of Expanded Program on Immunization in Pakistan has not been up to the mark, despite the fact that, achievement of universal immunization in the country remained a priority national agenda since long; yet, the success achieved is below par, and much work is still to be done^55,56^. There has been huge amount of funding into the Polio Eradication Initiative of EPI program in Pakistan; yet, few years back the coverage worsened in some areas due to poor management^17^. It is believed by the public health experts that Polio eradication in Pakistan has mainly been driven by international pressure, rather than by the demands of local communities or health staff or management or by the political leadership. The recent surveys have shown improvements in EPI working and handling the situation in collaboration with its supporting organizations like WHO and UNICEF^21^, but there is still long journey to travel for the realisation of our health goals.

Our study had certain strengths to its credit. Firstly, this is the first ever study in Pakistan to discuss country-wide vaccination coverage data collected through EPI Pakistan, to our knowledge. Secondly, this is the first study to detect the spatiotemporal variation in EPI vaccination coverage in Pakistan at the district level and explored the low vaccination coverage districts for all the antigens used in routine immunization program in the country. Thirdly, the methods used in this study are simple yet robust and are easy to understand for the readers as well as decision makers. Lastly, our study provided scientific evidence on low vaccination coverage clusters which transcended districts and provincial administrative boundaries so that the policy makers can make decisions, targeting clusters with low vaccination rates.

There were a few limitations in this study as well. Firstly, the data in the study was provided by EPI National cell and may be biased as it was collected by their own staff throughout the country. Secondly, the demographic details of individual vaccinated children (e.g., age, sex, home address etc.) were not available and hence not used in our study. Thirdly, the health staff involved in vaccination activities in different districts had different levels of skills, expertise and motivation and this may have been masked in the finalized data. Fourthly, the retrospective extrapolation of the population at a constant rate of 2.4% across all the districts is a limitation as the population growth in all the districts do not follow a uniform pattern, yet it was the best option as the growth rates for individual districts were not available. Lastly, the data did not reflect vaccination coverage in urban and rural populations separately.

## Conclusion

Our study detected low vaccination coverage clusters for each type of EPI vaccination in Pakistan at the district level, and has helped us understand and visualize immunization trends in Pakistan over 5 years. The desired success in getting rid of the burden of vaccine preventable diseases and improving child health cannot be achieved without attaining homogeneous levels of vaccination coverage throughout the country. The low vaccination coverage districts in FATA, KPK and Balochistan are a major concern; however, at the same time Punjab and Sindh with relatively higher vaccination coverage should not be considered risk free. The efficiency of EPI at national, provincial and district level needs to be enhanced. Decision makers, e.g., political leadership and the public health experts need to design in-depth research to probe the low-vaccination coverage in detail and also should amend the existing policies and implementation strategies to better the current situation.

## Methods

### Data collection

The district level, vaccination coverage data for each antigen included in the routine immunization program in Pakistan from 2012 to 2016 were collected from the Federal EPI Islamabad Pakistan. Immunization services are delivered to the community by more than 7,000 EPI centres throughout the country with each centre serving an average population of 30,000 individuals approximately. Immunization services are delivered to the community through fixed sites as well as by mobile or outreach approaches^26^. The data on vaccination and its coverage is reported from sub-district level to district level, and then to the respective provinces and eventually reaches the Federal EPI via vaccine Logistics Management and Information System (vLMIS)^26^.

The data used in this study were from 142 of 146 districts in Pakistan. Two of the districts which were not included in our study (Frontier Bannu and Frontier Lakki Marwat) were from Federally Administered Tribal Areas (FATA). The non-inclusion of the data from these two districts was due to the fact that rest of the districts in FATA (other than these two; Frontier Bannu and Frontier Lakki Marwat) remained conflict struck during most period of the study^31^, and the public sector healthcare facilities from these two districts were designated as make-shift vaccination hub catering most of the vaccination load from neighbouring districts. As a result, in trying to compensate for the missed non-vaccinated children the achieved vaccination targets in these districts reached even 150% or sometimes around 200%, which could not be reflected in the data analysis and therefore were excluded. Besides these two districts, district named Panjpai as shown in the map (in Balochistan Province) is an aberration in the map being used for current analysis and no such district exist in Pakistan; also the data from Indian held Kashmir was neither available nor included in our study.

Population data for each of the districts in Pakistan were downloaded from Pakistan Bureau of Statistics’ official website depicting latest statistics from census completed in 2017^57^. As the last population census in Pakistan was done in 1998 and between these two censuses (1998 to 2017), the national average annual growth rate was estimated to be 2.4%. The 2017 district wise population was retrospectively extrapolated, depreciating by 2.4% annually and gave us the population for each of the preceding year in the study respectively (2016, 2015, 2014, 2013, and 2012). The provinces and administrative units in Pakistan are shown in the supplemental file S1 for the reference purpose (for further demographic information on provinces in Pakistan see under the heading, “Methods” and sub-heading, “Study area” in the following link: https://www.ncbi.nlm.nih.gov/pmc/articles/PMC6025434/)59.

### Data analysis

#### Data organization, descriptive analysis and sequence plots

The raw vaccination coverage data by type, obtained from Federal EPI Pakistan were organized in Microsoft Excel as per the requirements of spatial analysis. EPI Pakistan calculates the target population of the eligible children for vaccination on the basis of WHO criteria and the average national population growth rate of the country^58^. Children less than 1 year of age are estimated to be 3.5% of the total population which was taken as target population by EPI for the vaccination coverage in Pakistan^26^. The sub-district level health staff on some occasions has been found over-reporting the vaccination coverage data to meet their performance indicators^7^ and the in-migrations between the districts were also not accounted for in the 3.5% target population. These two factors were considered to produce bias in the reported numbers of vaccinated children. To control this bias, we increased the target population by 1%; i.e., the target population in our study was set to be at 4.5% rather than 3.5%. The explanation for this adjustment is that, variation in the vaccination coverage in Pakistan reported in the literature has been between 40 and 80% (mean of which is 60% i.e. maximum reported vaccination coverage is reduced by 20%). The average difference between vaccination rates of BCG for 3.5% and 4.5% target population respectively for all the districts for each study year was calculated to be (19 to 21%) ~ 20%. Thus, the bias in our data was minimized by increasing the target population by 1% (3.5% to 4.5%) and the administrative data rates were reduced on the average by 20% for all types of vaccination included in this study.

The annual vaccination rate (VR) per 100 children, separately for each year and for each type of vaccination (BCG, OPV3, Penta3 and Mealsles1) were calculated at the district level. The descriptive statistics for vaccination rate of each type of vaccination were calculated for all 5 years of study separately (2012 to 2016). All the calculated summary statistics included mean values, standard deviation, minimum and maximum values, and quartiles of 25, 50 and 75 respectively.

Sequence plots for each type of vaccination coverage were plotted with years on the X-axis and vaccination rates by type on the Y-axis at the provincial levels and displayed in figure. The descriptive statistics and sequence plots were performed using statistical package SPSS 13.0^59^.

### Spatiotemporal analysis

GeoDa 1.8 software, a GIS tool used for creating and utilizing maps, discovering and sharing geographic information, compiling geographic data and analysing mapped information, was applied to create maps of spatiotemporal pattern of vaccination coverage^60^.

For spatial cluster analysis, the already organized data from Microsoft Excel sheet containing annual vaccination coverage data by type and district wise annual population and coordinates of centroids (latitudes and longitudes) were then run in the SaTScan 9.4.4^61^. SaTScan is applied to detect spatial clusters of diseases and for distribution of resources like interventions against any specific diseases^28^. SaTScan has also been used to detect the spatial heterogeneity in the vaccination coverage in the field of epidemiology.

In current study, for the detection of low-rates of vaccination coverage by type, we performed purely spatial scanning analysis using the discrete Poisson model for the districts’ population at risk. SaTScan has the provision of performing Poisson based modelling for the number of events in a fixed interval of time in any geographical setting having non-random and unique coordinates (assigned by researcher). The underlying spatial heterogeneities are also adjusted for the population while running this discrete Poisson model in SaTScan. To avoid overlapping of primary clusters by the secondary ones, selection of “No geographic overlap” was done as a default setting. Initially, we performed scan statistics using different settings of maximum radii for detection of low coverage clusters (e.g. 100 km, 200 km, 300 km and 400 km), and 5%, 10% and 15% for maximum population at risk and ultimately the final parameters were selected having cluster radii limit of 200 km and the limit for spatial cluster size to be for maximum 10% of the population at risk. Another spatial cluster study had adopted the same settings criteria using SaTScan software^62^. By using these finalized settings, primary and secondary clusters of low vaccination coverage by type were identified. Primary clusters were the most statistically significant clusters with low vaccination coverage; while, the secondary clusters were additional statistically significant clusters with low vaccination coverage by type in each year from 2012 to 2016.

The spatial patterns of the low vaccination coverage by type exhibited by their respective vaccination coverage rates (percentage) and all the derived information from SaTScan (information on primary and secondary clusters of low vaccination coverage by type, 2012–2016) were then displayed via maps using GeoDa 1.8 software.

### Ethical clearance

For this study, vaccination coverage by type was obtained from Federal EPI Cell via letter no. {F.No EPI/Gen/1-2016} dated 9th Feb 2018. An exemption approval letter was obtained from National Bioethical Committee Pakistan via letter number No.4-87/NBC-279-Exempt./17/1139. The research protocol was approved by Institutional Review Board of Xi’an Jiaotong University, China.

## Supplementary information

Supplementary file1 (PDF 308 kb)

## Data Availability

The Federal EPI Cell, provided vaccination coverage data and took undertaking from the authors to use these data exclusively for this study. As, we are not authorized to share it, so, the data is unavailable to access.

## Abbreviations

BCG: Bacillus Calmette Guerin
DTP: Diphtheria tetanus pertussis
EPI: Expanded program on immunization
FATA: Federally Administered Tribal Areas
GIS: Geographical Information System
KPK: Khyber Pakhtunkhwa
NADRA: National Database Authority Pakistan
OPV: Oral polio vaccine
PDHS: Pakistan Demographic and Health Survey
PSLM: Pakistan social and living standards measurement survey
UNICEF: United Nations Children Fund
VR: Vaccine rate
vLMIS: Vaccine Logistics Management Information System
WHO: World Health Organization
