# Supplementary material for: Spatiotemporal clustering analysis of Expanded Program on Immunization (EPI) vaccination coverage in Pakistan
Source: Sci Rep. 2020 Jul 3;10:10980. doi: 10.1038/s41598-020-67839-0 (PMC7335212; doi:10.1038/s41598-020-67839-0)
Supplement: Supplementary file 1 — Supplementary file1 (PDF 308 kb) [file 41598_2020_67839_MOESM1_ESM.pdf]

Spatiotemporal clustering analysis of Expanded Program on Immunization (EPI) vaccination coverage in Pakistan

Muhammad Farooq Umer [1,2], Shumaila Zofeen [1] Wenbiao Hu [3], Xin Qi [1,4,*] and Guihua Zhuang [1,*]

[1] School of Public Health, Xi'an Jiaotong University Health Science Center, Xi'an 710061, China; rafooq@hotmail.com (M.F.U.); shumailazofeen@yahoo.com (S.Z.)

[2] Contech International Health Consultant, 2-G, Model Town, Lahore 54700, Pakistan

[3] School of Public Health and Social Work, Queensland University of Technology, Kelvin Grove, QLD 4059, Australia; w2.hu@qut.edu.au

[4] Global Health Institute, Xi'an Jiaotong University Health Science Center, Xi'an 710061, China

* Correspondence: chestertsee@outlook.com (X.Q.); zhuanggh@mail.xjtu.edu.cn (G.Z.);
Tel.: +86-29-8265-5108 (X.Q.); Tel.: +86-29-8265-5103 (G.Z.)

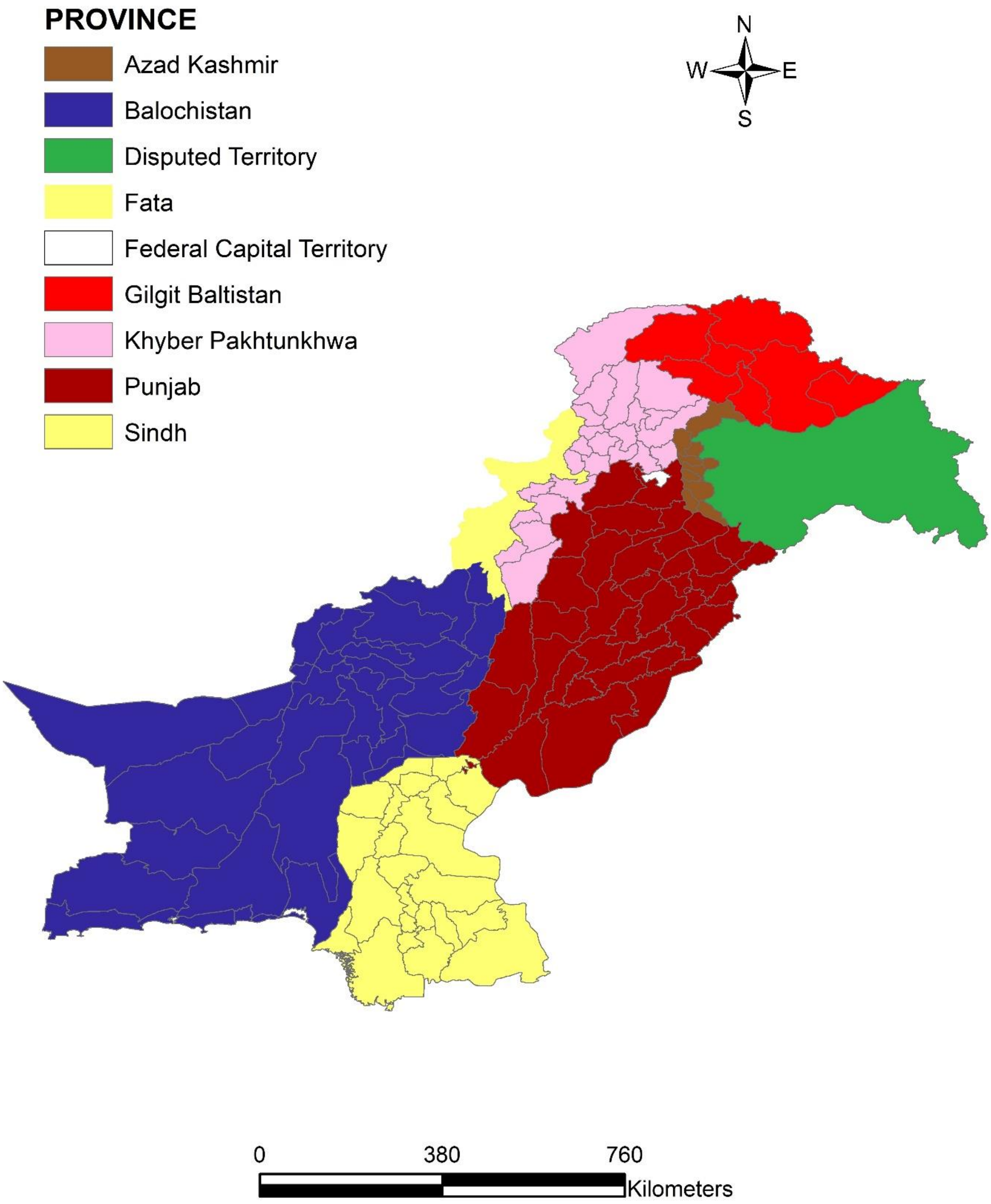
PROVINCE
Azad Kashmir
Balochistan
Disputed Territory
Fata
Federal Capital Territory
Gilgit Baltistan
Khyber Pakhtunkhwa
Punjab
Sindh
N
W
E
S
0
380
760
Kilometers
